# Spark Mapping Analysis for Segregation Partitioning in Large-Scale Super-Critical-Power Steel

**DOI:** 10.3390/ma18133128

**Published:** 2025-07-01

**Authors:** Baibing Li, Lei Zhao, Liang Sheng, Jingwei Yang, Liangjing Yuan, Lei Yu, Qiaochu Zhang, Haizhou Wang, Yunhai Jia

**Affiliations:** 1 National Center for Materials Service Safety, University of Science and Technology Beijing, Beijing 100083, China; d202310580@xs.ustb.edu.cn; 2 Central Iron and Steel Research Institute, Beijing 100081, China; zhaolei@ncschina.com (L.Z.); shengliang91@163.com (L.S.); yuanliangjing@ncschina.com (L.Y.); yulei@ncschina.com (L.Y.); zhangqiaochu@ncschina.com (Q.Z.); 3 NCS Jiangsu Testing Technology Co., Ltd., Suzhou 215300, China; yangjingwei@ncschina.com

**Keywords:** composition distribution, partition analysis, P91 steel, segregation, SMALS

## Abstract

The material properties of P91 steel, a critical high-temperature heat-resistant steel, are critically dependent on the uniformity of its macro-composition distribution. This paper presents the first application of Spark Mapping Analysis for Large Samples (SMALS) for the non-destructive, full-field characterization of macro-composition distribution in P91 steel ingots and finished tubes. To address the analytical challenges posed by large-sized specimens, an innovative partition-based statistical analysis model was developed, enabling the effective demarcation of large-scale macro-segregation areas. Utilizing Sample A as the paradigm, a systematic methodology and workflow for the partition analysis were established, successfully identifying and quantifying the widths of its positive and negative segregation bands (namely 6 mm, 20 mm, and 8 mm). This approach was subsequently applied to samples from different smelting batches (B1, B2, C1, C2), effectively delineating macro-segregation areas within each sample and performing quantitative evaluations based on the statistical upper segregation limit. The findings provide essential experimental insights into the full-field compositional heterogeneity of P91 steel and deliver critical methodological guidelines for optimizing steel smelting processes to control and mitigate macro-segregation.

## 1. Introduction

Improving thermal power generation technology is one of the essential means to enhancing the energy efficiency and achieving energy saving and emission reduction [1]. The thinning of the central steam pipe wall thickness and high-temperature reheat steam pipe will play a key role in saving resources. P91 steel has good high-temperature durability and has a wide range of applications as a high-temperature and high-pressure heat-resistant steel material [2,3]. It is widely used in sub-critical and super-critical thermal power generation units of the main steam pipe and high-temperature reheat steam pipe, showing excellent overall performance [4]. For large steel ingots, segregation significantly compromises mechanical and physical properties, potentially inducing anisotropy, reducing yield strength, impairing product performance, and ultimately diminishing the service performance and lifespans of steel products, thereby introducing potential safety hazards [5]. Therefore, analyzing the composition distribution of P91 steel pipe facilitates addressing issues such as creep damage, pinhole cracks, and thermal fatigue damage on the inner surfaces of in-service high-temperature components.

Selective crystallization readily occurs during solidification, inducing solute precipitation at the solidification front. When elemental concentrations in steel increase significantly and diffusion within the liquid phase becomes inadequate, a high-concentration precipitation layer forms at the solidification front, thereby inducing segregation. The segregation distribution of most steel components is typically sampled by multi-hole drilling and analyzed using techniques such as Inductively Coupled Plasma Optical Emission Spectrometry (ICP-OES) [6]. Although ICP-OES demonstrates high detection capability for steel samples, the analysis of large-scale samples necessitates drilling multiple holes at designated locations followed by chemical dissolution. This labor-intensive procedure compromises the accurate representation of surface component distribution. Furthermore, the method is cumbersome, time-consuming, and unsuitable for analyzing the distribution of the C element [7]. However, the carbon (C) content in steel largely determines critical properties such as strength, plasticity, and toughness, making the comprehensive analysis of C essential.

Laser-Induced Breakdown Spectroscopy (LIBS) offers significant advantages for compositional distribution analysis due to its elimination of sample preparation requirements [8]. For instance, Quackatz et al. utilized LIBS to visualize chemical distributions on corroded duplex steel surfaces, validating the technique’s accuracy through comparative analysis with conventional methods such as X-ray energy-dispersive spectroscopy (EDS) [9]. Similarly, Kimura et al. employed X-ray diffraction (XRD) for direct texture measurement in boiler tube cross-sections, observing the influence of the steel surface on macro-segregation [10]. Their findings indicate that reducing macro-segregation enhances the creep strength and highlight XRD’s utility for non-destructive steel evaluation. However, LIBS exhibits limitations for metal analysis, primarily stemming from lower reproducibility and heightened susceptibility to matrix and self-absorption effects.

Micro-beam X-ray fluorescence (*μ*-XRF) is an essential non-destructive technique for elemental composition analysis in steel. Sheng studied high-speed railway axle steel samples using Spark Mapping Analysis for Large Samples (SMALS), comparing results with *μ*-XRF [11]. While both methods demonstrated concordance for high-concentration elements, SMALS exhibited superior precision in quantifying trace-level constituents. Although XRF can analyze samples that are up to 200 mm long, it has low sensitivity for Al analysis, limiting its utility for some metallic elements. Consequently, no suitable and efficient analytical method currently exists for large steel castings. Conventional methods require cutting samples into smaller pieces, preventing the comprehensive analysis of full components.

**Figure 1:**
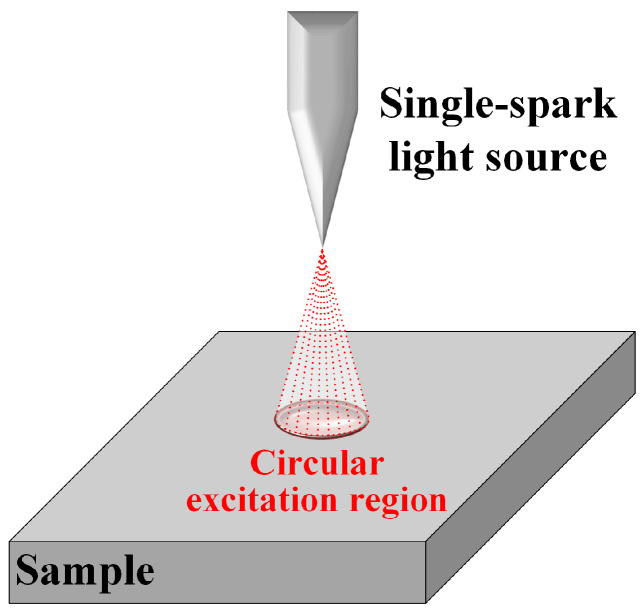
Schematic diagram of single-spark light source excitation.

Figure 1 presents a schematic diagram of a single-spark light source. The self-developed high-stability continuous-discharge spark light source delivered outstanding performance, with the ability to operate continuously for over 17 h.

This single-spark light source facilitates prolonged elemental composition distribution analysis for large-scale samples. Unlike conventional sources, it eliminates pre-ignition requirements and preserves material surface integrity, thereby ensuring the acquisition of unaltered compositional data. The system circumvents signal distortion inherent to pre-ignition processes and, when integrated with continuous excitation and high-speed acquisition technologies, enables bulk data collection within a single operation cycle. This configuration significantly accelerates analytical throughput while reducing processing times.

Coupled with a precision positioning system, the light source permits the precise localization of material defects. Through the statistical analysis of multiple single-discharge signals, it provides high-resolution insights into elemental heterogeneity, advancing the quantitative assessment of material homogeneity.

The technology supports extensive surface scanning, yielding comprehensive inclusion distribution profiles and representative analytical outcomes. By contrast, conventional sources are restricted to localized analyses, impeding the holistic characterization of inclusion dispersion.

This approach resolves critical challenges in characterizing large metallic components—including high-speed train wheels/axles, aircraft engine turbine disc blanks, and nuclear/ultra-supercritical thermal power plant pipelines—by enabling the full-area mapping of compositional segregation and inclusion concentrations.

Building upon the developmental research of this instrumentation system, we have extended its applications and established a partitioning framework incorporating statistical methodologies. This framework enables both macro-scale segregation delineation and characteristic zone assessment in large-scale components. Specifically, SMALS was employed to develop the partition analysis method using a P91 steel pipe (sample A). The validated methodology was subsequently implemented across P91 billets and pipe sections from Batches B1, B2, C1, and C2. During steel solidification, elements undergo positive and negative segregation. Standards exist to specify acceptable ranges for elemental composition in steel samples, ensuring proper melt processing. Therefore, for maximum positive and negative segregation zones in selected steel elements, there must be an upper limit defining maximum segregation for different steel grades. This upper limit determines the material’s conformity level, expressed as the upper limit of the segregation degree $D_{s(\max)}$. The upper limit of the segregation degree $D_{s(\max)}$ of heat-resistant steel was evaluated by Zhang [12]; Equation (1) is as follows, where *C* is the element content in percentage:

(1) $$D_{s(\max)}=9.024C^{-0.451}$$

When samples were examined by SMALS, a large amount of data was collected from the scanning area. The positive segregation degree $D_{s(+)}$ and negative segregation degree $D_{s(-)}$ of the sample were calculated by Equations (2) and (3) as follows, in which $C_{97.5\%}$ and $C_{2.5\%}$ were the contents in normal distribution at the right and left sides for the 95% confident level. $\bar{\bar{C}}$ was the general mean content.

(2) $$D_{s(+)}=\frac{C_{97.5\%}-\bar{\bar{C}}}{\bar{\bar{C}}}\times 100\%$$

(3) $$D_{s(+)}=\frac{C_{2.5\%}-\bar{\bar{C}}}{\bar{\bar{C}}}\times 100\%$$

## 2. Materials and Methods

### 2.1. Equipment

**Figure 2:**
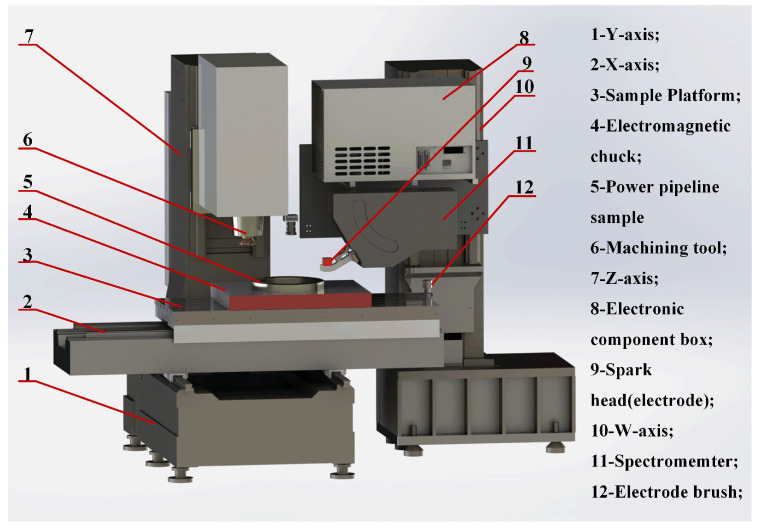
Schematic setup of the SMALS equipment.

Figure 2 schematically illustrates the SMALS system configuration, accommodating samples up to 1000 mm × 500 mm in planar dimensions. The sample surface was milled by a vertical numerical control machine prior to scanning. Prior to scanning, sample surfaces underwent precision milling via a vertical computer numerical control (CNC) machine. The vertical machining system employed a rigid sample stage with 900 kg maximum load capacity. Samples were secured on this stage, enabling the integrated processing center to execute controlled *Z*-axis milling operations perpendicular to the surface plane. The sample was scanned and excited by a digital spark source along the *X*-axis at a traverse speed of 1 mm/s. The milling process was cooled by compressed air with water and oil filtration. The milling speed was 500 r/min, the feed rate was 300 mm/min, the milling depth was 0.1 mm, the parallelism of the *X*-axis and the *Y*-axis was 0.01 mm, and the roughness Ra < 3.2 um. This instrument represents the first-of-its-kind integration of historical processing data with spark detection technology. The system achieves continuous integration of sample processing, analytical measurement, and spectral characterization, eliminating manual handling while preventing secondary contamination and repositioning errors. This tripartite architecture enables automated component processing, precision scanning localization, and quantitative spectral analysis within a unified platform.

**Figure 3:**
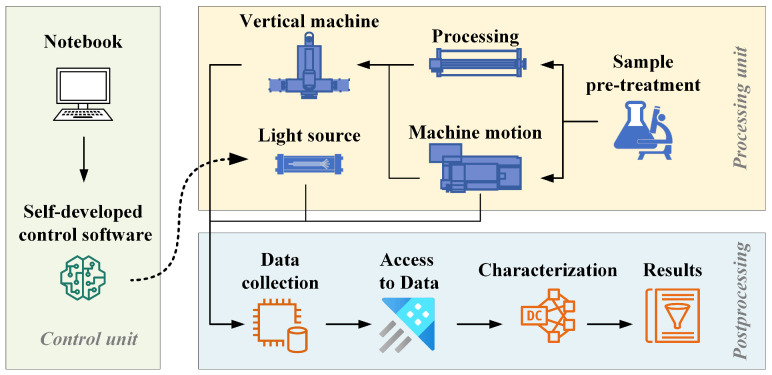
SMALS system workflow diagram.

The SMALS serves as an analytical instrument designed to acquire high-resolution, continuous compositional information at the meter scale. This integrated system comprises six principal subsystems: a fully automated sample processing system, a Roland Park optical system, a spark light source system, a laser light source system, a stacked grating system, and a data processing system. The operational workflow of the SMALS is depicted in Figure 3.

Following sample preparation, spectral acquisition parameters and scanning method are configured. The Paschen-Runge Optical System’s Photomultiplier tube (PMT) detector with a wavelength range of 160–650 nm is used to scan and excite the sample with an all-digital, solid-state spark light source. Precise alignment of the sample stage and excitation assembly precedes programming of stage motion trajectories and calibration of constant source-to-surface distance throughout the scanning sequence. Empirical evidence from multiple experimental trials indicates that maintaining a 0.04 mm gap between the excitation assembly and sample surface optimizes spark excitation efficiency. Upon completion of positional calibration, the control software automatically edits the motion-test G code and sends the test command. The electrode material of the spark excitation source discharges single-spark events during scanning of the prepared sample. The sample scanning mode can be customized according to the geometric coordinates of diverse sample shapes. The instrument employs horizontal pre-ignition followed by unidirectional scanning along the *X*-axis. The initial pre-ignition phase requires a 4 s dwell time at the commencement of each scan line, synchronized with contact between the sample stage and the sample surface. Initiating at the commencement of each scanning line, the initial interface between the instrument’s spark stage and the sample surface undergoes a 4 s pre-ignition phase to stabilize the spark excitation parameters. Subsequently, spark-scanning excitation is conducted across the sample surface at a constant scanning velocity of 1 mm/s. The instrument systematically traverses the sample in 4 mm wide swaths, enabling comprehensive analysis and detection of macroscopic samples through sequential top-to-bottom and left-to-right area scanning.

Following spectral acquisition by the spectrometer assembly, the photomultiplier tube (PMT) detection system concurrently records multi-element spectral intensity signals. These signals are transmitted to the computational unit via the data acquisition interface, where specialized software converts intensity data into elemental concentrations. As illustrated in Figure 1, this conversion employs a calibration function relating spectral intensity ratios to concentration ratios, mathematically formalized in Equation (4) [13].

For the *i*-th measurement, *R_i_* is defined as the spectral line intensity ratio, with *I_a_*_,*i*_ and *I_r_*_,*i*_ being the intensities of the analytical and reference lines, respectively. *C_i_* represents the elemental concentration at the location of the corresponding *i*-th single discharge signal.

(4) $$R_{i}=I_{a,i}/I_{r,i}=K∙C_{i}^{b}$$

### 2.2. Samples

**Figure 4:**
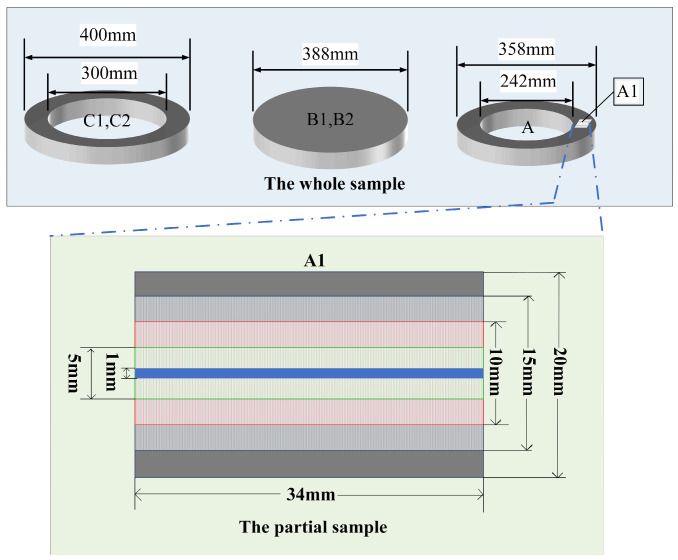
Schematic diagram of steel samples A, B1, B2, C1, and C2.

The P91 series samples depicted in Figure 4 include sample A, sourced from one factory for establishing the partition analysis method, and samples B1, B2, C1, and C2, derived from another factory for verifying and applying this method, all from the same batch. B1 and B2 were sectioned from billet B while C1 and C2 were obtained from seamless steel pipe C, which was fabricated by piercing and rolling billet B. Sample A exhibited an outer diameter of 358 mm and an inner diameter of 242 mm; a subset, denoted as A1, measured 34 mm in length by 20 mm in width. B1 and B2 constituted top and bottom segments of a steel round billet with a diameter of 388 mm whereas C1 and C2 represented top and bottom rings of diameter 388 mm, extracted from the steel pipe. These five samples underwent scanning and examination via SMALS. Comprehensive sample specifications are detailed in Table 1, with rings defined by outer diameter (OD), inner diameter (ID), and thickness (T) and billets indicated by the symbol φ (diameter).

### 2.3. Calibration Curve

As presented in Table 2, calibration employed over twenty certified reference materials (CRMs) of stainless steel. In the process of constructing the calibration curve, multiple certified reference materials (steel standards) encompassing the elemental concentration range of the test specimens were subjected to excitation and corresponding spectral data were collected. The content ranges (%) of the selected standard reference materials for each specific element are detailed in Table 2. The analytical wavelengths utilized were 212.41 nm (Si), 293.30 nm (Mn), 267.71 nm (Cr), 218.49 nm (Ni), 281.61 nm (Mo), and 233.01 nm (Cu). All CRMs underwent surface preparation via grinding with 60-mesh silicon carbide (SiC) abrasive paper. Calibration curves demonstrated excellent linearity, with coefficients of determination (R^2^) exceeding 0.99 for all elements.

### 2.4. Partition Analysis Method

Samples were scanned along the *X*-axis direction (left to right). For samples A, C1, and C2, a standoff distance of 10 mm was maintained from inner ring edges and 14 mm from outer ring edges to preclude excitation effects. Similarly, round billets (B1, B2) employed a 14 mm outer margin standoff. Crucially, metallurgical products exhibited symmetrical characteristics in both cross-sectional and longitudinal sectional analyses. During steel solidification [14], segregation localization and severity are predominantly governed by the cooling rate and the inherent characteristics of each alloying element. Given that segregation patterns mirror material symmetry, annular partitioning—extending radially from the inner to outer edges—was implemented for cross-sectional analysis of round billets and steel pipes. This partition-based segregation analysis constitutes an effective methodology for large-scale metallic specimens. The statistical framework was established using sample A as the methodological benchmark, subsequently validated, and applied to Specimens B1, B2, C1, and C2. This standardized approach enables consistent examination of additional samples.

The steps of the partition analysis method are as follows:

(1) Measuring the shape and size of steel samples and completing the excitation scanning by SMALS.
(2) Setting the partition statistics gradients of round sample and naming these areas as area I, area II, area III, etc. along the center of the circle toward the outer edge.
(3) Collecting the data of the analyzed area after setting the gradient and evaluating the boundary of the segregation according to the trend of the area mean concentration.
(4) Evaluating the degree of segregation with the upper limit and the statistical fitting degree by the 95% criterion.

The segregation is classified according to the deviation of the original average alloy concentration $\bar{\bar{C}}$ from the solute concentration $C_{s}$ in each alloy part, where $C_{s}>\bar{\bar{C}}$ is positive segregation and $C_{s}<\bar{\bar{C}}$ is negative segregation [15]. Following sample scanning, the mean value across all samples is calibrated against the known average actual content. Consequently, under conditions of mass conservation where the global average remains invariant, macroscopic segregation manifests as compensatory variations across distinct regions—specifically, elevated concentrations in segregated areas counterbalance depleted concentrations elsewhere.

## 3. Results and Discussion

### 3.1. The Fluctuation Effect on the Different Scanning Areas

**Figure 5:**
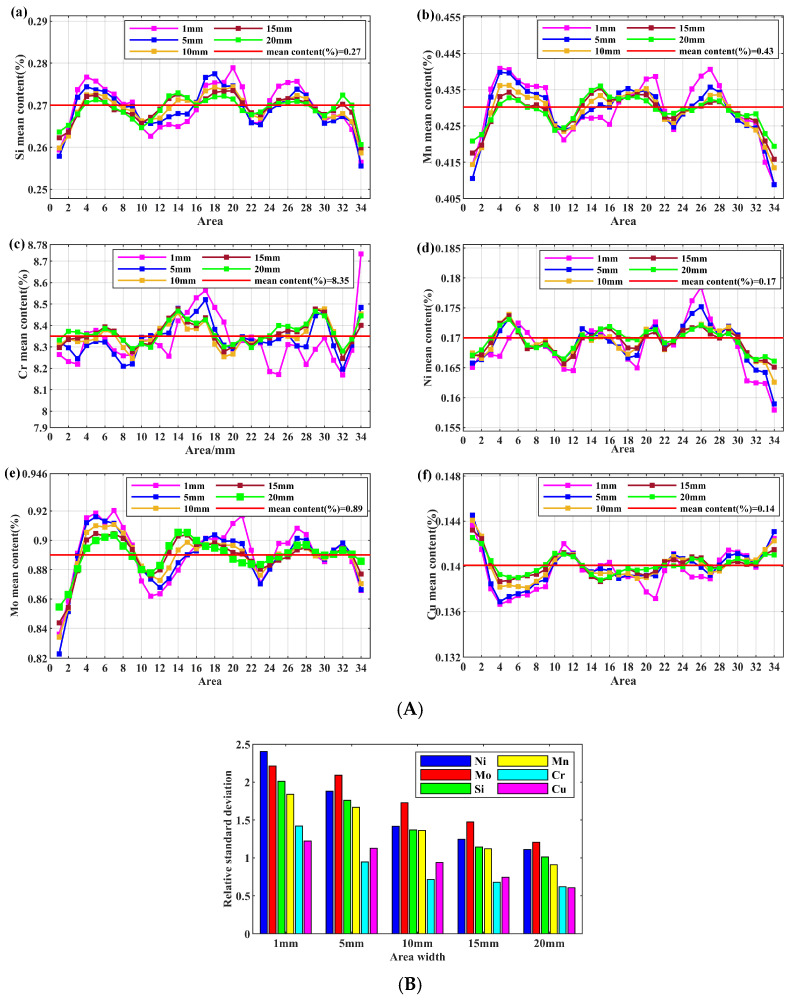
(**A**) Statistical map of the content fluctuations with different area width in (**a**) Si, (**b**) Mn, (**c**) Cr, (**d**) Ni, (**e**) Mo, and (**f**) Cu. (**B**) Diagram of relative standard deviation with different area width for elements.

For sample A1, discrete scanning fields of approximately 1 mm, 5 mm, 10 mm, 15 mm, and 20 mm width (all maintaining a constant 34 mm length) were evaluated. Scanning was performed continuously at a firing rate of 1 mm/s, with a corresponding density of 125 firing points per square millimeter. Consequently, increasing the scan width proportionally elevated the data volume; accordingly, each scan area was subdivided into 34 sub-areas for independent calculation of its average content, as illustrated in Figure 5A. For scanning fields of 1 mm, 5 mm, 10 mm, 15 mm, and 20 mm widths, the corresponding data volumes across 34 partitioned sub-regions were 125, 625, 1250, 1875, and 2500 measurement points, respectively. Given that localized sampling lacks representativeness for holistic elemental composition analysis, the overall partition statistics method was employed. This approach not only accounts for spatial distribution along all axes but also provides critical recommendations for optimizing the solidification process of metals. The six elements—Si, Mn, Cr, Ni, Mo, and Cu—were subjected to statistical analysis to examine fluctuations in mean content across sub-regions under varying area widths. It was observed that as the area width increased, the amplitude of content fluctuations progressively diminished, converging toward the general mean content for each element.

As illustrated in Figure 5B, the statistical analysis of the relative standard deviation (RSD) across varying area widths revealed an inverse correlation: increased area width corresponded to a systematic reduction in the RSD. This trend indicates that larger sampling areas mitigate the impact of minor systematic errors, thereby enhancing the reliability of macroscopic segregation distribution studies. Furthermore, elemental segregation analysis exhibited scale-dependent characteristics, necessitating distinct analytical approaches at different observational scales. When analyzing large-scale samples, we abandoned the conventional approach of localized sub-region selection and instead implemented a concentric radial partitioning mechanism. This methodology maximizes both the stability of spatial representation and the analytical accuracy in assessing macro-scale segregation phenomena. For investigating macroscopic segregation in large-scale samples, such as steel pipes or ingots, the element-rich or element-depleted central zones are typically situated at a certain distance from the sample boundaries. To ensure curve accuracy and mitigate issues arising from excessive regional subdivision, the initial samples were partitioned into ten regions. Subsequently, a more statistically robust area width for macroscopic segregation analysis was determined based on the convergence zones between element-rich and element-depleted areas. It is noteworthy that the criteria for area division differed between rings and billets. Specifically, the element-enriched region narrowed significantly following ring perforation, necessitating a corresponding reduction in the designated area width. Consequently, the development of this partitioned statistical methodology for large-scale samples was critically important for the accurate analysis of compositional distribution.

### 3.2. Data Collection and Analysis of Complete Samples

**Figure 6:**
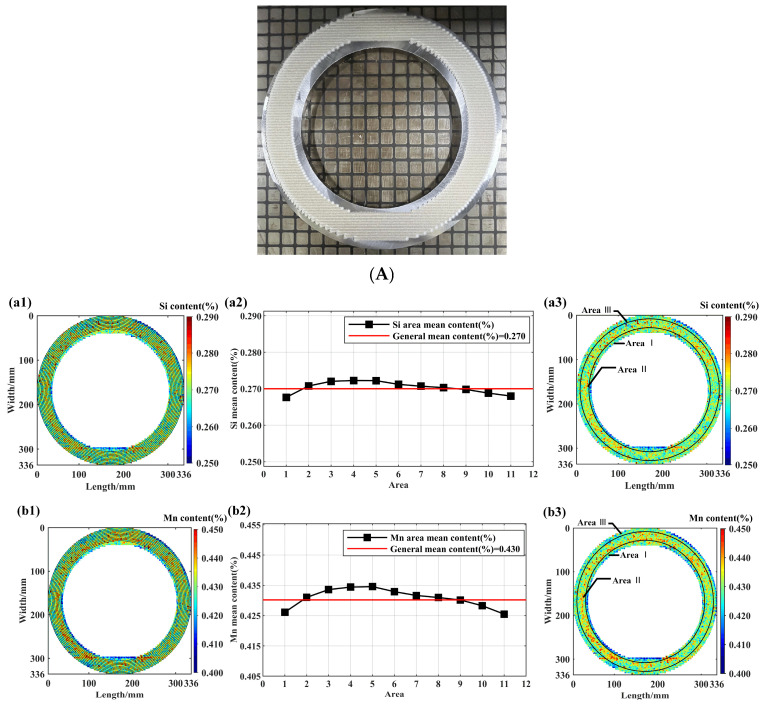

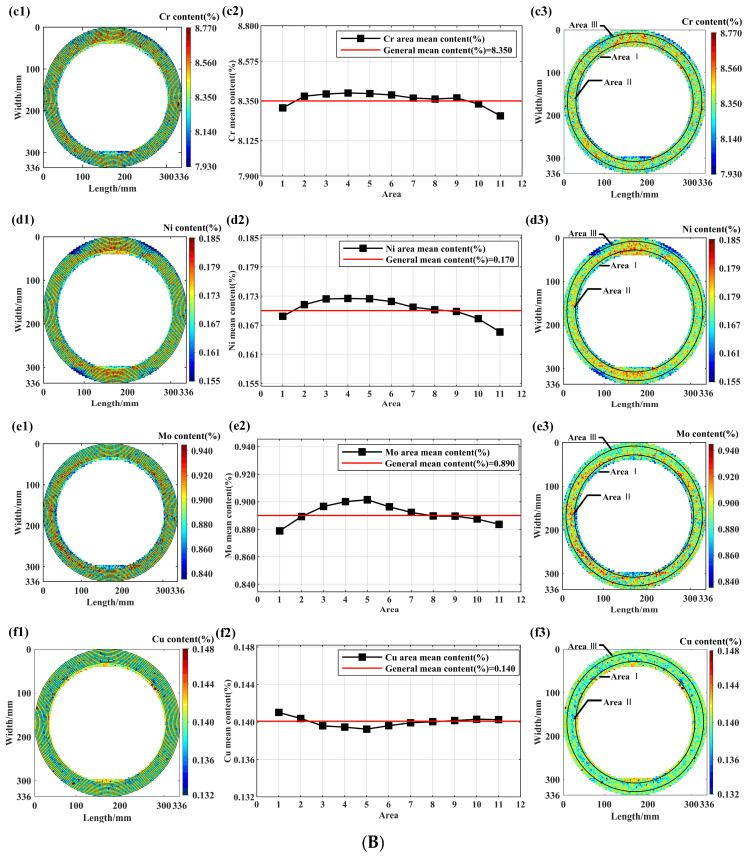
(**A**) Diagram of sample A scanned by SMALS. (**B**) Statistical map of the scanned partition for sample A for the elements Si, Mn, Cr, Ni, Mo, and Cu, (**a1**–**f1**) Element scans from center to edge (11 areas); (**a2**–**f2**) Area mean content; (**a3**–**f3**) Final areas partitioned based on statistical significance (vs. general mean content).

Figure 6A depicts the physical sample A undergoing scanning. Figure 6B(a1–f1) present the scanned data extracted at equidistant intervals (3 mm) from the ring center to the outer edge, resulting in the delineation of 11 distinct areas. Subfigures (a2), (b2), (c2), (d2), (e2), and (f2) display the average concentrations of various elements calculated within these 11 independent areas; the abscissa denotes the uniformly partitioned areas while the ordinate represents the mean elemental content within each area. Finally, subfigures (a3), (b3), (c3), (d3), (e3), and (f3) illustrate the definitive partitioned areas determined through statistical comparison between the regional mean content and the overall global mean content. As depicted in Figure 6B(a2–f2), critical concentration transitions for the six elements predominantly occurred within the second and ninth zones. Consequently, sample A was partitioned into segments measuring 6 mm, 20 mm, and 8 mm, reflecting a similar segregation trend exhibited by elements Si, Mn, Cr, Ni, Mo, and Cu. Specifically, the concentration profiles of Si, Mn, Cr, Ni, Mo, and Cu delineated three distinct regions extending radially from the inner to the outer edge. Elements Si, Mn, Cr, Mo, and Ni demonstrated a negative–positive–negative segregation pattern whereas Cu exhibited a positive–negative–positive pattern. This differential segregation behavior is attributed to the higher heat extraction rate at the sample edges compared to the interior, establishing a significant temperature gradient within the ingot [16]. Elemental distribution varied across different sections of the ingot, particularly between the periphery adjacent to the mold and the interior regions. The enhanced cooling rate at the periphery relative to the core impeded complete elemental diffusion within the liquid phase. Consequently, undiffused elements accumulated locally, forming positive or negative compositional segregation areas. Within the macroscopic segregation process, inter-dendritic liquid flow opposed the solidification direction, inducing negative segregation. Considering the respective melting points—Cu (1083 °C) versus Si (1414 °C), Mn (1246 °C), Cr (1907 °C), Ni (1455 °C), and Mo (2623 °C)—low-melting-point elements became enriched in the central casting region. This enrichment arose from shrinkage-induced cavity formation between dendrites during solidification, generating localized negative pressure. Concurrent temperature decline further promoted gas precipitation from the liquid, establishing a pressure differential. Consequently, element-enriched liquid migrated along intercolumnar channels toward the higher-pressure casting periphery. Crucially, ring-shaped steel specimens enabled the precise delineation of positive and negative segregation zones, thereby facilitating the targeted analysis of these distinct regions in full-scale industrial samples.

Table 3 is a statistic table of component segregation of different areas in sample A. The positive and negative segregation areas, area mean content, positive segregation degree, negative segregation degree, statistical fitting degree, and standard deviation of different elements are counted.

The statistical fitting degree is the ratio of the position content in the specification range to the total content of all positions in the analyzed area [12]. All data outliers were evaluated according to the 95% fitting degree criteria because the confidence level in the chemical analysis was usually at the 95% confidence degree, so the statistical fitting degree had to be at least 95%. According to Table 3, it was found that the statistical fitting degree of all areas for sample A was greater than 95%. The positive and negative segregation degree of the steel samples did not exceed the upper limit $D_{s(\max)}$ for all areas of all elements, except for the positive segregation degree $D_{\left(+\right)}$ of element Cr in area I. The element content standard deviation and segregation were lower in the area, and their average values were higher than the general average for sample A. This was due to the fact that the elemental composition in the negative segregation areas did not diffuse uniformly. The excessive differences between the high and low elemental compositions were shown in the negative segregation areas. Thus, the element-enriched positive segregation area was more uniform.

### 3.3. Application of Partition Statistics to Other Steel Samples

**Figure 7:**
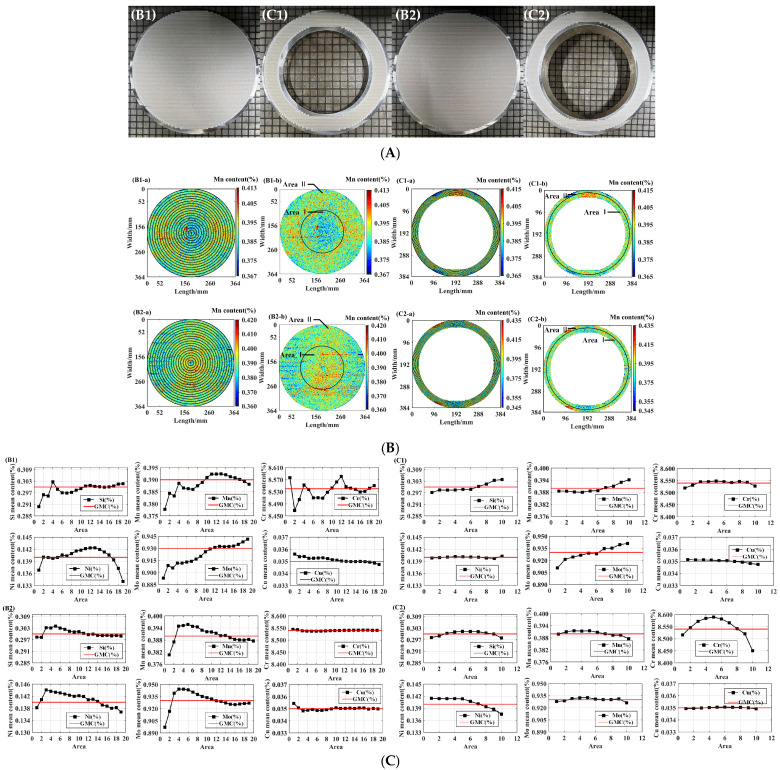
(**A**) Diagram of the steel samples scanned by SMALS. (**B**) Elemental segregation map of the Mn element. (**C**) Statistical map of the scanned partition of the steel sample.

The partitioned statistical methodology demonstrated applicability to both annular and cylindrical specimens. Systematic radial measurements originating from the geometric center toward the periphery enabled the precise delineation of segregation zones. Figure 7 presents cross-sectional scanning analyses of super-critical-power steel components: a (A) top cylindrical billet, (B1) upper annular section, (C1) bottom cylindrical billet, (B2) lower annular section, and (C2) complementary annular profile.

Figure 7B shows the two-dimensional plots of Mn elemental contents about the partitioning process and partitioning completion in the four samples. Take the Mn element as an example: the partition statistics of each sample were set to the same gradient, and the partition gradient of the billet was set to 10 mm; this could be divided into 19 areas, as shown in Figure 7B(B1-a,B2-a); The ring sample was a part of the finished steel pipe, and the 3 mm excitation area of the scan was divided into ten areas, as shown in Figure 7B(C1-a,C2-a).

According to this partitioning gradient, Figure 7C(B1,B2,C1,C2) shows the statistical graphs of the average content of each area of the elements Si, Mn, Cr, Ni, Mo, and Cu in the four samples; GMC is the general mean content. The top sample B1 and the bottom sample B2 of the round billet showed that most of the elements started to rise above the total average or fall below the total average around the ninth area; the top ring C1 and the bottom ring C2 showed that most of the elements started to rise above the total average or fall below the total average around the fourth area. Therefore, as in Figure 7B(B1-b,B2-b), the samples were divided into two areas from 90 mm to the outer edge of the billet, and as in Figure 7B(C1-b,C2-b), the ring was also divided into two areas from 18 mm to the outer edge.

The content fluctuations of samples B1 and B2 were higher than those of C1 and C2 because the steel pipe was pierced from the billet. In the piercing process [17], the heating temperature of the billet should be controlled in the best deformation temperature range; the average heating temperature is generally controlled at 1200–1250 °C, and then, after stress relief annealing, normalizing, tempering, straightening, grinding, and other processes, the final pipe is formed. Therefore, some defects are eliminated during the processing of real steel pipes, so the fluctuations of the pipe were relatively minor compared with the billets in this study. From Figure 7C(B1,B2), it was found that most of the elements had positive and negative segregation separation circles whose half diameter values were about 1/2 of the sample half diameter. From Figure 7C(C1,C2), it was found that most of the elements had positive and negative segregation separation circles at a position close to 2/3 of the sample ring thickness. The partitioning statistics methods of segregation area for round and ring samples were also applicable to other steel samples.

For samples B1 and C1, the area I of Si, Mn, Cr, Ni, and Mo was the negative segregation area, and area II was the positive segregation area. The Cu element in area I was of positive segregation, and area II was of negative segregation. For sample B2, the Cr elements in areas I and II were uniform. The area I of Si, Mn, Ni, and Mo was of positive segregation; the area II was of negative segregation. The area I of the Cu element was of negative segregation and area II was of positive segregation. In the bottom ring C2, the area I was of positive segregation and area II was of negative segregation for most elements.

Partition statistics were performed for the top round billet B1, the top ring C1, the bottom round billet B2, and the bottom ring C2, and the data are shown in Table 4. For samples B1 and B2, the positive and negative segregation degree were evaluated by the upper limit D_S(max)_. Al elements in sample B1 and B2 did not exceed the upper limit D_S(max)_ in areas I and II, except that the segregation degree of Cr in sample B1 was slightly exceeded. As shown in Figure 7C, the fluctuation of Cr content in sample B1 was higher than that in sample B2. Hence, the positive and negative segregation degrees of B1 were also higher than those of sample B2. It can be seen that the statistical fitting degree of all elements in areas I and II was satisfied with over 95% requirement. For samples C1 and C2, all elements did not exceed the upper limit D_S(max)_ in area I and II, except that the segregation degrees of Cr in sample C2 and Mo in sample C1 were slightly exceeded. It can be seen that the statistical fitting degree of all elements in area I and II was satisfied with over 95% requirement, except for the element Mo in sample C1. Table 4 shows that the element content standard deviation and segregation were lower in the area where the average value was higher than the general average for sample P91.

## 4. Conclusions

This study systematically delineated the structural configuration, operational workflow, and innovative features of the Scanning Macro-Analysis Laser System (SMALS), proposing a partitioned statistical methodology to extend its applicability to large-meter-sized specimens. Implemented on P91 steel across cylindrical billets and annular sections, this framework enables the extraction and interrogation of localized compositional datasets from full-field scans, facilitating direct comparison between regional and bulk characteristics, individualized zone-specific diagnostics, and the targeted selection of characteristic domains within heterogeneous distributions. These advancements deepen the mechanistic understanding of segregation phenomena while establishing new protocols for macroscopic-material characterization.

(1) The rule of partition statistics is based on comparing the area mean content with the general mean content; the area means lower than the general mean belong to one area and the area means that are higher belong to another. The positive and negative segregation areas of the steel samples were clearly separated by the partition method. With sample A, the partition statistics method was established to determine the segregation areas, and the positive and negative segregation degree and the statistical conformity degree were examined for large samples. The distribution of elements could be seen from a macro perspective, which was more comprehensive than the analysis of small areas.
(2) The partition-based statistical method was applied to assess elemental distribution in round billets and corresponding pipe rings, with concentric regions defined by radial distance from the geometric center. Billets were segmented into inner and outer zones (outer zone: 90 mm radius) while pipe rings were partitioned into dual regions (outer zone: 18 mm radius). Statistical analysis revealed consistent elemental distribution trends between both forms, demonstrating that the piercing process significantly mitigated segregation in pipe rings—evidenced by attenuated compositional fluctuations and enhanced homogeneity in finished pipes relative to initial billets.
(3) Utilizing the Spark Mapping Analysis for Large Samples (SMALS) technique, the entire surface of large samples was scanned to map the elemental composition distribution of P91 steel. Analysis revealed that in areas exhibiting an average element content exceeding the overall mean for the same P91 pipe, the standard deviation of element content and segregation was significantly lower. This finding implies that element-rich regions display reduced variability in composition compared to element-poor zones. During solidification, attributable to differences in melting points, copper (Cu), a low-melting-point element, manifested an inverse diffusion trend relative to other elements in solute diffusion processes.

## Figures and Tables

**Table 1:** Sample specification and elemental content.

Sample	Specification (mm)	Elemental Content (%)					
		Si	Mn	Cr	Ni	Mo	Cu
A	358 × 242 × 58	0.27	0.43	8.35	0.17	0.89	0.14
B1	*ϕ*388	0.30	0.39	8.54	0.14	0.93	0.035
C1	400 × 300 × 50						
B2	*ϕ*388						
C2	400 × 300 × 50						

**Table 2:** Calibration curve of elements Si, Mn, Cr, Ni, Mo, and Cu.

Element	Spectral Lines (nm)	Content Range (%)	Calibration Curve (Rounded to Two Significant Figures)	R^2^
Si	212.41	0.103~1.05	$0.00035\times I^{2}+0.027\times I-0.053$	0.994
Mn	293.30	0.13~1.96	$0.010\times I^{2}+0.0066\times I-0.0019$	0.993
Cr	267.71	7.4~24.1	$1.39\times I^{2}-0.21\times I-0.000039$	0.992
Ni	218.49	0.062~1.43	$0.015\times I^{2}+0.010\times I-0.0019$	0.997
Mo	281.61	0.089~1.01	$-0.015\times I^{2}+0.043\times I-0.011$	0.991
Cu	233.01	0.061~0.69	$0.0018\times I^{2}+0.0012\times I-0.00087$	0.999

**Table 3:** Statistic table of component segregation of different areas in sample A.

Elements	General Mean Content (%)	Area	Area Mean Content (%)	DS (−) (%)	DS (+) (%)	DS (Max) (%)	Statistical Fitting Degree (%)	Specification Range (%)	Standard Deviation (%)
Si	0.270	I	0.268	−4.45	5.79	16.61	100.00	0.20~0.50	0.0080
		II	0.271	−3.55	4.62		100.00		0.0060
		III	0.269	−4.06	5.10		100.00		0.0070
Mn	0.430	I	0.427	−3.76	4.24	13.47	100.00	0.30~0.60	0.0090
		II	0.433	−2.92	3.65		100.00		0.0080
		III	0.427	−3.61	3.65		100.00		0.0080
Cr	8.350	I	8.325	−3.31	3.68	3.53	98.98	8.00~9.50	0.15
		II	8.381	−2.93	3.18		99.89		0.13
		III	8.309	−3.45	3.39		98.26		0.15
Ni	0.170	I	0.169	−5.27	6.22	20.47	100.00	≤0.40	0.0049
		II	0.172	−4.89	5.00		100.00		0.0043
		III	0.167	−8.13	5.93		100.00		0.0057
Mo	0.890	I	0.881	−4.16	4.34	9.70	95.38	0.85~1.05	0.019
		II	0.895	−3.61	4.20		99.72		0.018
		III	0.886	−3.47	3.66		98.87		0.016
Cu	0.140	I	0.141	−2.65	2.81	22.34	99.98	≤0.20	0.0031
		II	0.140	−2.48	2.43		100.00		0.0023
		III	0.140	−2.34	2.29		99.98		0.0028

**Table 4:** Statistical table of component segregation of different areas in samples B1, C1, B2, and C2.

Element	General Mean Content (%)	Sample	Area	Mean Content (%)	DS (−) (%)	DS (+) (%)	DS (Max) (%)	Statistical Fitting Degree (%)	Specification Range (%)	Standard Deviation (%)
Si	0.300	B1	I	0.298	−3.87	6.52	15.84	99.96	0.20~0.40	0.0087
			II	0.301	−3.41	4.57		100.00		0.0062
		C1	I	0.298	−8.95	9.59		100.00		0.014
			II	0.302	−9.31	10.90		100.00		0.015
		B2	I	0.302	−3.72	6.32		100.00		0.0077
			II	0.299	−3.81	6.68		99.98		0.0084
		C2	I	0.301	−3.62	3.73		100.00		0.0057
			II	0.300	−4.36	4.26		99.98		0.0074
Mn	0.390	B1	I	0.388	−3.77	4.52	14.07	100.00	0.30~0.50	0.0083
			II	0.391	−3.47	3.89		100.00		0.0074
		C1	I	0.392	−6.99	8.35		99.99		0.015
			II	0.392	−6.99	8.35		99.99		0.015
		B2	I	0.393	−4.36	5.03		100.00		0.0094
			II	0.389	−4.42	5.20		99.99		0.01
		C2	I	0.391	−3.78	4.10		100.00		0.0078
			II	0.389	−4.55	4.17		99.99		0.0086
Cr	8.540	B1	I	8.524	−4.65	5.8	3.50	99.47	8.00~9.50	0.23
			II	8.545	−4.52	5.37		99.68		0.22
		C1	I	8.540	−2.11	2.35		100.00		0.097
			II	8.539	−2.07	2.33		100.00		0.096
		B2	I	8.538	−0.11	0.10		100.00		0.0047
			II	8.541	−0.11	0.10		100.00		0.0051
		C2	I	8.571	−3.96	4.50		99.89		0.18
			II	8.508	−5.27	4.64		98.36		0.21
Ni	0.140	B1	I	0.141	−4.57	4.66	22.34	100.00	0~0.20	0.0033
			II	0.140	−10.05	5.99		100.00		0.0053
		C1	I	0.140	−3.14	3.18		100.00		0.0023
			II	0.140	−2.95	2.97		100.00		0.0021
		B2	I	0.143	−11.34	12.05		100.00		0.0085
			II	0.139	−12.09	12.49		99.99		0.0088
		C2	I	0.141	−7.16	9.37		100.00		0.0059
			II	0.139	−10.63	8.56		99.99		0.0065
Mo	0.930	B1	I	0.917	−3.22	3.82	9.51	100.00	0.85~1.05	0.017
			II	0.934	−2.96	3.38		100.00		0.015
		C1	I	0.924	−11.24	11.05		91.28		0.053
			II	0.938	−12.4	13.22		89.05		0.062
		B2	I	0.938	−3.25	3.33		100.00		0.016
			II	0.927	−3.09	3.50		99.99		0.016
		C2	I	0.931	−4.28	4.58		99.98		0.021
			II	0.929	−4.25	4.67		99.94		0.021
Cu	0.035	B1	I	0.0352	−1.87	1.72	41.74	100.00	0.00~0.10	0.00032
			II	0.0349	−2.02	1.80		100.00		0.00034
		C1	I	0.0351	−4.56	4.53		100.00		0.00082
			II	0.0349	−5.57	4.85		100.00		0.00092
		B2	I	0.0349	−1.93	9.51		100.00		0.00080
			II	0.0350	−1.85	7.43		100.00		0.00078
		C2	I	0.0350	−1.26	1.24		100.00		0.00022
			II	0.0350	−1.37	1.29		100.00		0.00024

## Data Availability

The original contributions presented in this study have been included in the article. Further inquiries can be directed to the corresponding authors.

## References

[1] Rerak M., Ocłoń P. (2017). Thermal Analysis of Underground Power Cable System. J. Therm. Sci..

[2] Pandey C., Mahapatra M.M., Kumar P., Saini N. (2018). Some Studies on P91 Steel and Their Weldments. J. Alloys Compd..

[3] Han K., Ding H., Fan X., Li W., Lv Y., Feng Y. (2022). Study of the Creep Cavitation Behavior of P91 Steel under Different Stress States and Its Effect on High-Temperature Creep Properties. J. Mater. Res. Technol..

[4] Maddi L., Ballal A.R., Peshwe D.R., Mathew M.D. (2020). Influence of Normalizing and Tempering Temperatures on the Creep Properties of P92 Steel. High Temp. Mater. Process..

[5] Ge H., Ren F., Li J., Hu Q., Xia M., Li J. (2018). Modelling of Ingot Size Effects on Macrosegregation in Steel Castings. J. Mater. Process. Technol..

[6] Zheng P., Luo Y., Wang J., Yang Y., Hu Q., Mao X., Lai C. (2022). Improved Solution Cathode Glow Discharge Micro-Plasma Source with a Geometrically Optimized Stainless Steel Auxiliary Cathode for Optical Emission Spectrometry of Metal Elements. Microchem. J..

[7] Adya V.C., Sengupta A., Thulasidas S.K., Natarajan V. (2016). Direct Determination of S and P at Trace Level in Stainless Steel by CCD-Based ICP-AES and EDXRF: A Comparative Study. At. Spectrosc..

[8] Liu R., Rong K., Wang Z., Cui M., Deguchi Y., Tanaka S., Yan J., Liu J. (2020). Sample Temperature Effect on Steel Measurement Using SP-LIBS and Collinear Long-Short DP-LIBS. ISIJ Int..

[9] Quackatz L., Griesche A., Kannengiesser T. (2022). Spatially Resolved EDS, XRF and LIBS Measurements of the Chemical Composition of Duplex Stainless Steel Welds: A Comparison of Methods. Spectrochim. Acta Part B At. Spectrosc..

[10] Kimura K., Kwak K., Nambu S., Koseki T. (2020). Nondestructive Evaluation of Macro Segregation in Creep Strength Enhanced 9Cr–1Mo–V–Nb Steel. Scr. Mater..

[11] Sheng L., Yuan L., Jia Y., Zhao L., Zhang X., Yu L., Zhang Q., Wang H. (2022). Full-Scale Spark Mapping of Elements and Inclusions of a High-Speed Train Axle Billet. J. Anal. At. Spectrom..

[12] Zhang X., Jia Y., Sheng L., Yuan L., Li J. (2022). Characterization of Segregation Degree for Large Size Metal Component and Application on High-Speed Train Wheel. Anal. Chim. Acta.

[13] Wang H., Zhao L., Jia Y., Li D., Yang L., Lu Y., Feng G., Wan W. (2020). State-of-the-Art Review of High-Throughput Statistical Spatial-Mapping Characterization Technology and Its Applications. Engineering.

[14] Li J., Xu X., Ren N., Xia M., Li J. (2022). A Review on Prediction of Casting Defects in Steel Ingots: From Macrosegregation to Multi-Defect Model. J. Iron Steel Res. Int..

[15] Shen Y., Yang S., Liu J., Liu H., Zhang R., Xu H., He Y. (2019). Study on Micro Segregation of High Alloy Fe–Mn–C–Al Steel. Steel Res. Int..

[16] He Q., Wu H., Meng H., Hu Z., Xie Z. (2019). Molten Steel Level Detection by Temperature Gradients With a Neural Network. IEEE Access.

[17] Zhang Y., Yi R., Wang P., Fu C., Cai N., Ju J. (2021). Self-Piercing Riveting of Hot Stamped Steel and Aluminum Alloy Sheets Base on Local Softening Zone. Steel Res. Int..

